# The Effect of Vitamin E and Metallothionein on the Antioxidant Capacities of Cadmium-Damaged Liver in Grass Carp* Ctenopharyngodon idellus*

**DOI:** 10.1155/2018/7935396

**Published:** 2018-11-04

**Authors:** Yang Feng, Xiaoli Huang, Yajiao Duan, Wei Fan, Jing Duan, Kaiyu Wang, Yi Geng, Ping Ouyang, Yongqiang Deng, Defang Chen, Shiyong Yang

**Affiliations:** ^1^Department of Aquaculture, College of Animal Science & Technology, Sichuan Agricultural University, WenJiang 611130, Sichuan, China; ^2^Neijiang City Academy of Agricultural Sciences, Neijiang 641000, Sichuan, China; ^3^College of Veterinary Medicine, Sichuan Agricultural University, Wenjiang 611130, Sichuan, China; ^4^Sichuan Provincial Agricultural Department, Chengdu 613000, Sichuan, China

## Abstract

Cadmium (Cd) causes a broad spectrum of toxicological effects to animals. Aquatic animals were more likely to accumulate Cd than terrestrial animals because of the living environment. Clearance of Cd in aquatic animals has become an important part of aquatic food safety. The present study was focused on the oxidative damage induced by Cd in the liver of grass carp* Ctenopharyngodon idellus* and the protective effect of vitamin E (VE) and metallothionein (MT). Grass carp were divided into four groups: the control group, Cd+phosphate-buffered saline (PBS) group, Cd+VE group, and Cd+MT group. All fish were injected with CdCl_2_ on the first day and then VE, MT, and PBS were given 4 days after injection, respectively. The liver function and antioxidant capacity of grass carp were evaluated. Cd administration resulted in damage of liver function and morphology in liver, which was expressed as the increased content of AST and ALT, rupture of organelles, and decrease of CAT, SOD, and GSH-Px activity. However, VE and MT treatments protected against Cd-induced damage of liver in grass carp by decreasing AST and ALT content, repairing organelles, and maintained the antioxidant system by elevating CAT, SOD, and GSH-Px activity and regulating related mRNA transcript expression. The results revealed that VE and MT might play an important role in the treatment of heavy metal poisoning through their antioxidative effects.

## 1. Introduction

Cadmium (Cd) could endanger human and animal health by causing multiple organ damage, which may result in prostate, lung, and testes cancer [1] and kidney, liver, bone, and brain injury, as well as immune and cardiovascular system impairment [2]. However, due to the special living environment, aquatic animals were more threatened by Cd than terrestrial animals [3]. Cd carried in aquatic animals could be delivered to humans indirectly through the food chain and endanger human health. This issue is critical to Cd intoxication research, not only for the aquaculture industry but also for human health.

Reactive oxygen species (ROS), peroxides, superoxide, hydroxyl radical, and singlet oxygen, for example, are chemically reactive chemical species containing oxygen. It could not only cause damage to the antioxidant system in cells, but also induce endoplasmic reticulum and mitochondrial stress, urging autophagy and unfolded protein response (UPR) [4, 5] and causing cells mortality ultimately [6, 7]. Reports found that when suffering from Cd damage, the antioxidant system of animals would be damaged, and a large number of reactive oxygen species (ROS) would be generated and attack the polyunsaturated fatty acids in the cell membrane thereby [8–10]. It then causes lipid peroxidation and changes the composition and structure of cell membranes, causing irreversible oxidative damage. In 1983, Muller first discovered lipid peroxidation in liver cells treated with Cd [11]. Though Cd does not catalyze the ROS by itself, it can indirectly generate ROS through various pathways, such as neutralizing the glutathione, affecting iron metabolism, interfering the energy metabolism, and uncoupling oxidative phosphorylation [12–14]. As an important detoxified organ in fish, liver was generally treated as the richest accumulation position of heavy metal [15]. It may be also the most damaged organ caused by oxidative damage of Cd.

Metallothionein (MT) and vitamin E (VE) have an important role in scavenging ROS and stabilizing the cell membranes [16, 17]. Cd can often induce the activation of the endogenous protective mechanism of the body, promote the up-regulation of endogenous MT, and protect the antioxidant system [18, 19]. However, the amount of MT produced by the body induced by Cd is limited, which cannot avoid the invasion of Cd [20, 21] but leads us to think about the anti-Cd effect of exogenous MT in the body. VE will be used to compare the antioxidant capacity of exogenous MT in this study. Previously, we had found that exogenous VE and MT could effectively reduce the Cd-caused malondialdehyde (MDA) in liver of grass carp [22]. Nevertheless, whether they could recover the antioxidant capacity and protect the damaged liver under Cd challenging is still unknown. In order to make it clear how exogenous VE and MT perform their antioxidant protection on Cd-induced liver damage, an “intoxication-detoxication” pattern of grass carp has been established. Liver function, hepatic ultrastructure changes, antioxidase levels, and the transcriptional expression of related genes were detected in the present research.

## 2. Materials and Methods

### 2.1. Drugs and Fishes Preparation

Purified cadmium chloride (CdCl_2_) was obtained from MOLBASE (Shanghai) Biotechnology Co. Ltd. VE (liposoluble; purity 99%) and MT-2 (pure rabbit liver MT powder; purity 99%) were purchased from Sigma-Aldrich Company (Beijing, China) and Shanghai Shou Feng Industrial Co. Ltd., respectively. All other chemicals were of analytical grade or the highest grade available and obtained from local companies. CdCl_2_, VE, and MT were dissolved in sterilized phosphate-buffered saline (PBS), and VE was emulsified using an ultrasonic crusher until milky.

Healthy grass carp juveniles (weight: 50 ± 3.4 g, length: 15 ± 2.5 cm) were obtained from a commercial fish farm in Sichuan, China. The fish was acclimatized to the laboratory and fed with commercial pellets (Tongyi Company, Suzhou, China) twice a day for 2 weeks before experimentation. The carps were exposed to a light:dark cycle of 12 h:12h, a uninterrupted oxygen supply to ensure more than 5 mg/L dissolved oxygen, pH of 6.5–8.5, ammoniacal nitrogen, and nitrite maintained at 0–0.02mg/L. The water in the tank was pretreated with UV light and an aeration process, and 20% of the culture water was renewed every day. Fish that have a bright body color and are responsive, robust, and healthy were selected for experimentation. All animal handling procedures were approved by the Animal Care and Use Committee of Sichuan Agricultural University, following the guidelines of animal experiments of Sichuan Agricultural University, under permit number DY-S20144657.

### 2.2. Experimental Treatment

**Figure 1:**
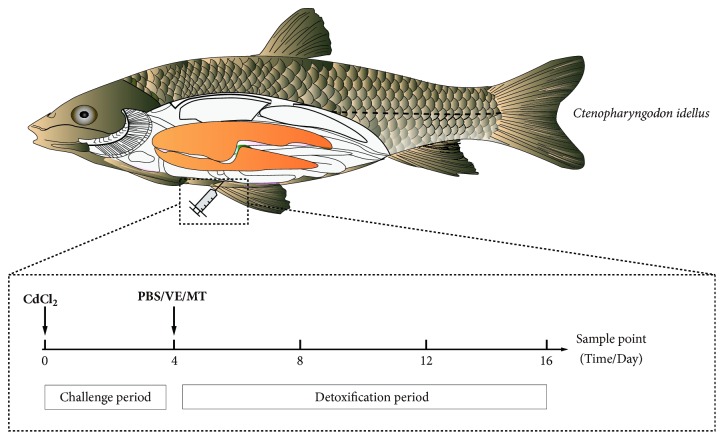
Study design showing the challenge model. Each group included 150 fishes divided into three parallel tanks.

Healthy fish (n=600) were randomly divided into 4 groups: the control group, Cd+PBS group, Cd+VE group, and Cd+MT group, and each group included three parallel tanks. The experimental cycle included two periods: the challenge period and the detoxification period (Figure 1). Lethal concentration (LC_50_) of CdCl_2_ was determined using 6 different concentrations (70, 102, 145, 207, 295, and 420 *μ* mol/kg) by an acute toxicity test, and the LC_ 50_ was 199.631 *μ*mol/kg according to the modified Kobvgguffer method [22, 23]. 20 *μ* mol/kg CdCl_2_ (1/10 LC50) as the subacute concentration in the present research was injected into each fish of first three groups intraperitoneally during the challenge period [24]. The group which was not given CdCl_2_ but injected with PBS was treated as controls. On the 4th day after Cd injection, challenged fish was medicated with 4 mL/kg PBS, 2.1 mg/kg MT [25], and 20 IU/kg VE[26], respectively. Samples were collected after treatment of the 4th, 8th, 12th, and 16th days after challenge.

### 2.3. Sample Collection and Ultrastructural Examination

Experimental fish (n=6) in each group was sampled for ultrastructure examination. Liver tissues were fixed rapidly with 2.5% glutaraldehyde and postfixed in 2% veronal acetate-buffered osmium tetroxide. After dehydration in graded alcohol, the tissues were embedded in Araldite. The blocks were sectioned in a microtome with a glass knife. Sections, 6575 nm thick, were placed in uncoated copper grids. The sections were stained with uranyl acetate, and poststained with 0.2% lead citrate. The subcellular structure of liver was examined with Hitachi H-600 Transmission electron microscope (TEM) (HITACHI, Tokyo, Japan).

### 2.4. Measurement of Liver Function and Antioxidant Indexes in the Liver

During the experiment, 6 fishes in each group were randomly sampled every 4 day. The livers were removed immediately and homogenized with 0.65% physiological saline (4°C). The homogenate was centrifuged at 3500×g 4°C and the supernatant was used to detect different indicators in the liver. The total protein in the supernatant was determined by total protein quantification kit (NJJCBIO, Nanjing, China). The activity of superoxide dismutase (SOD), catalase (CAT), glutathione peroxidase (GSH-Px), aspartate aminotransferase (AST), and alanine aminotransferase (ALT) was detected using biochemical kits (NJJCBIO, Nanjing, China), following the manufacturer's protocol.

### 2.5. Gene Expression Levels of Antioxidase in the Liver

At 4, 8, 12, and 16 days, six fishes from each group were anesthetized by MS-222. Livers were sampled, placed in RNA/DNA protector solution (TaKaRa, Dalian, China), and stored at 4°C. Then the livers were homogenized by crushing with a mortar and pestle and stored at −80°C.

Total RNA was isolated from livers with TRIzol Reagent kit (TaKaRa, Kusatsu, Japan). Complementary DNA (cDNA) was synthesized from 1 *μ*g of RNA using the PrimeScript™ RT reagent kit with gDNA Eraser (TaKaRa, Kusatsu, Japan). qPCR was performed using an SYBR green real-time PCR kit (TaKaRa, Kusatsu, Japan) and a Thermo Cycler (BioRad, California, USA).* β-actin* and* 18S ribosomal RNA* were used as reference genes to determine the relative expression of target genes, which were invariant expression in grass carp liver in our previous validation. The primers used for qPCR are listed in Table 1.

For qPCR, the 25 *μ*L reaction mixture contained 12.5 *μ*L SYBR Green PCR Master Mix, 8.5 *μ*L diethylpyrocarbonate-treated water, 1.0 *μ*L of forward primer, 1.0 *μ*L of reverse primer, and 2 *μ*L cDNA. The following program conditions were used for the reactions: 3 min at 95°C for 1 cycle, samples were amplified for 40 cycles at 95°C for 10 s, melting temperature of a specific primer pair for 30 s, followed by 10 s at 95°C and 72°C for 10 s. To distinguish between specific and nonspecific reaction products, a melting curve was obtained at the end of each run. The 2^-ΔΔCT^ method was used to calculate relative changes in mRNA transcript expression from the qPCR results (ΔCT = CT_target  gene_ –CT_reference  gene_, ΔΔCT = ΔCT_experimental_ – ΔCT_control_) [27].

### 2.6. Statistical Analysis

The results are expressed as the mean value (n=6) and standard deviation. The significance of differences was analyzed by variance analysis. The analysis was performed using one way analysis of variance while the* t*-test was applied to determine whether the differences between groups were significant (SPSS v.20.0, IBM Corp., New York, USA). A value of* P* < 0.05 was considered significant, while a* P* < 0.01 was considered highly significant.

## 3. Results

### 3.1. VE and MT Maintain the Liver Function after Cd Challenged

**Figure 2:**
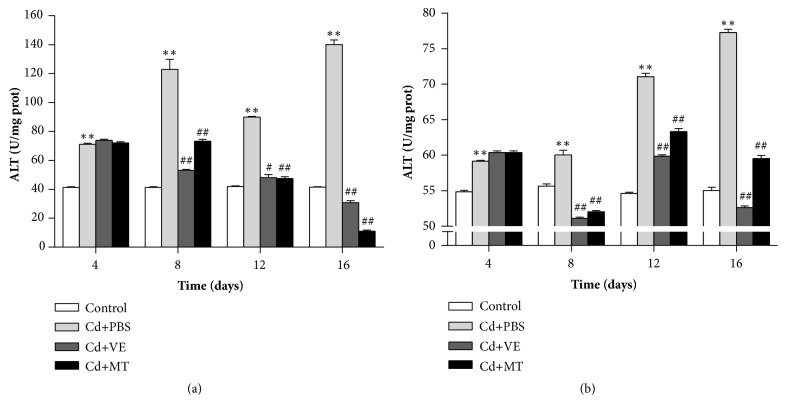
Assessment of AST and ALT in liver from the grass carp exposed to the Cd and detoxicants. Note: data are presented with the means ± standard deviation. *∗* 0.01<*P*<0.05 or *∗∗ P*<0.01 represents a significant difference or highly significant difference between the control group and the PBS group. # 0.01<*P*<0.05 or ##* P*<0.01 represents a significant difference or highly significant difference between the PBS group and the VE / MT group. n=6 for each group.

AST and ALT, two highly conservative indicators in liver, are commonly located in hepatic cytoplasm and would release into the circulation when hepatocytes necrotize [28–30]. In order to detect the protective effect of VE and MT on the liver, the activities of the two enzymes in different groups were examined. The results showed that Cd could elevate the content of AST and ALT obviously compared with the control group (Figures 2(a) and 2(b)). On the 4th day, the difference reached highly significant (*P* < 0.01). On the 16th day, Cd caused a 3-fold rise in AST and a 1.5-fold rise in ALT. However, both VE and MT could hasten the recovery of AST and ALT and maintain the normal level of both enzymes compared with the PBS group (Figures 2(a) and 2(b)). On the 8th day, the AST and ALT levels in the VE and MT groups showed highly significant decrease (*P *< 0.01) and gradually returned to normal levels on the 12th day. The results indicated that VE and MT could maintain the liver function of hepatic cells after Cd challenge.

### 3.2. VE and MT Protected the Morphological Integrity of Liver Cells

**Figure 3:**
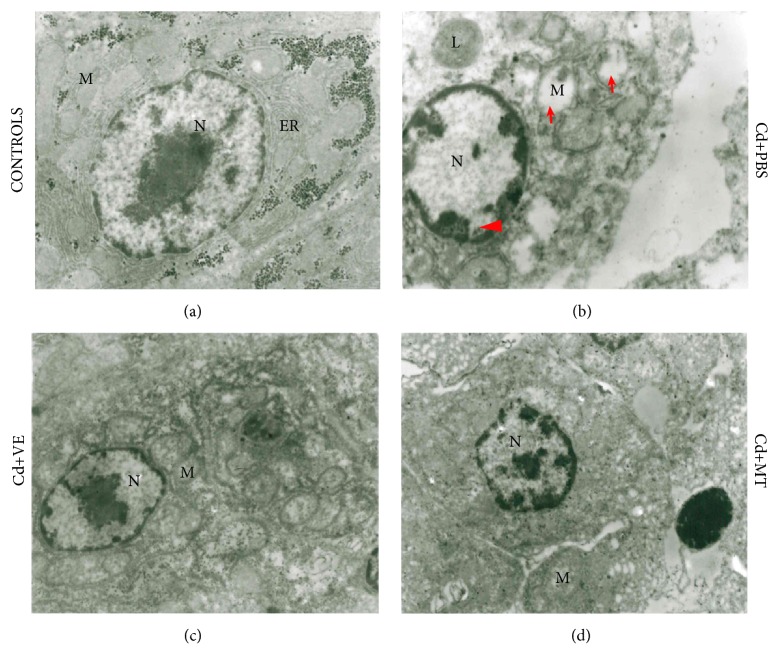
Ultrastructural pathology in the livers in different trails on 16th day. M: mitochondria; N: nuclei; ER: endoplasmic reticulum; L: lysosomes, ×10000. (a) The liver of the control group. (b) Ultrastructural changes from the liver in the PBS group showed mitochondria swollen and broken, mitochondrial cristae and the glycogen particles disappeared, and chromatin condensed. Arrowhead: chromatin condensation and nucleolar margination. Arrow: mitochondria enlargement and disintegration. (c)-(d) Ultrastructural changes in VE and MT groups showed mitochondria and chromatin density recovered slowly, and the cell was gradually returning to normal.

In control group, various organelles arranged neatly and there are no significant morphological changes in the hepatic cells (Figure 3(a)). However, after Cd challenged, the hepatic cells of grass carp appeared retrogressive characteristics, which manifested as chromatin condensed, lysosomes increased, and mitochondrial swelled, along with the disappearance of mitochondrial cristae and glycogen granules, and the disruption of endoplasmic reticulum (Figure 3(b)).

After treatment of VE and MT, the condensed chromatin recovered gradually, and the morphology and number of mitochondria were maintained at normal level (Figures 3(c) and 3(d)). In addition, a large number of glycogen granules began to deposit in the hepatic cells. These results indicated that VE and MT could maintain the morphological stability of hepatic cells.

### 3.3. Antioxidation Biochemistry Parameters in the Liver

So far, there still has a question how VE and MT maintain the morphology and function of liver cells during Cd challenging. References showed that Cd generally produces ROS indirectly and causes damage to organelles [12–14]. Previous studies have also found that Cd elevated the content of MDA in the liver of grass carp, and VE and MT maintained its balance [22]. Therefore, VE and MT may protect the liver through increasing the hepatic antioxidant capacity. In order to explore the protective effect of VE and MT on liver antioxidant system of grass carp; the activities of three antioxidases, CAT, SOD, and GSH-Px, were evaluated in different groups.

**Figure 4:**
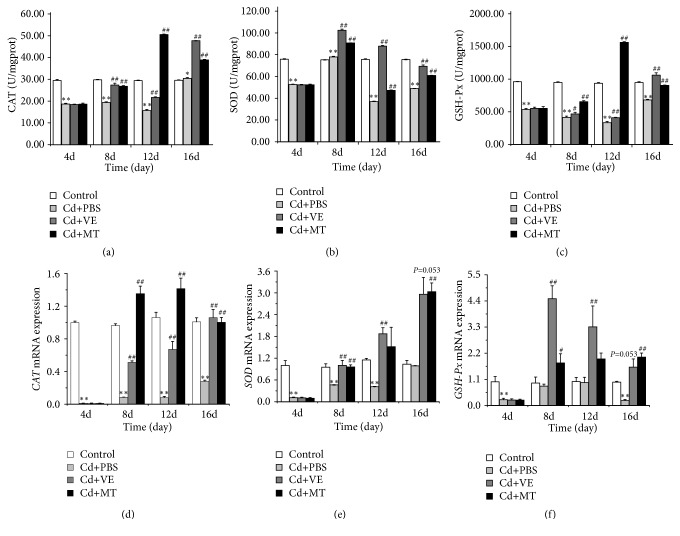
Assessment of antioxidases and expression of related genes in the liver of grass carp in different groups. Note: data are presented with the means ± standard deviation. *∗* 0.01<*P*<0.05 or *∗∗ P*<0.01 represents a significant difference or highly significant difference between the control group and the PBS group. # 0.01<*P*<0.05 or ##* P*<0.01 represents a significant difference or highly significant difference between the PBS group and the VE/MT group. n=6 for each group.

After Cd challenged, the activities of CAT performed significantly decrease (*P* < 0.01) (Figure 4(a)), which indicated that the antioxidant capacity of grass carp liver was attenuated due to the Cd-induced damage. VE and MT could recover the activities of CAT in grass carp, which reached at normal level on the 8th day already. On the 12th and 16th days, the CAT activity in the VE and MT groups was higher than that of control group. However, on the 16th day, the CAT level of the PBS group returned to normal levels that may be due to the self-healing ability of the liver.

Similarly, the activity of SOD and GSH-Px in the liver showed a highly significant decrease after Cd injection (*P* < 0.01), while VE and MT could recover the activity on some extent (Figures 4(b) and 4(c)). Among which, VE and MT could recover the activity of SOD on the 8th day, but the repair effect could not remain on the following time. Meanwhile, the two drugs could also recover the activity of GSH-Px on some extent after Cd challenging, but the values could not go back to normal compared with the control group. Moreover, two drugs showed some differences in recovery of various enzyme activities.

### 3.4. The mRNA Transcription of Antioxidation Related Genes

To investigate the mechanism of MT and VE in maintaining the activity of antioxidase, the mRNA transcription of* CAT*,* SOD*, and* GSH-Px* was examined.

After Cd challenged, the mRNA transcription of* CAT* performed significantly decrease (*P* < 0.01) (Figure 4(d)), which has dropped nearly 70-fold compare with that of the control group. On the 8th day, the mRNA transcription of* CAT* increased greatly in MT group and gradually returned to normal level. The result showed that MT might recover the CAT activity by elevating the mRNA transcription of* CAT*. Though VE could also recover the mRNA transcription of* CAT*, the ability was relatively weak.

The changes of mRNA transcription in* SOD* and* GSH-Px* were similar to* CAT*. Cd could decrease the mRNA transcription of* SOD* and* GSH-Px* significantly (*P* < 0.01) (Figures 4(e) and 4(f)). However, VE and MT could recover their transcription on 8th day significantly after Cd challenging (*P* < 0.01).

## 4. Discussion

Heavy metals had the potential to induce toxicological effects and led to the accumulation of ROS [31]. As lethal heavy metals in the environment, Cd could also stimulate the formation of ROS, thereby causing oxidative damage to tissues and resulting in the loss of the cell functions [8, 9, 32]. Therefore, we speculated that Cd could damage the body primarily through oxidative stress. In the present study, the ultrastructure pathology showed that Cd could damage the eliminated centre of ROS, the endoplasmic reticulum and mitochondria [33, 34]. Also, Cd induced severe oxidative stress response caused by Cd in grass carp liver, which showed a severe decrease in the activity of CAT, SOD, and GSH-Px and the transcription of related genes. Although there were emerging a rebound of the activities of antioxidases and the expression of related genes at the end of the experiment after Cd challenged, we speculated that it might be an adaptability of the organism which increased the ability of its own detoxification. In addition, it might also due to the fact that the reason for the Cd content of grass carp was gradually reduced after one-time injection of CdCl_2_ [35].

VE was first discovered by Evans and Bishop as an essential dietary factor for reproduction [36]. As the main lipid-soluble antioxidant in the body, VE plays an important role in scavenging free radicals and stabilizing cell membranes [37]. In addition, besides the chelation ability with metal ions [32, 38], MT also had excellent antioxidative effect [39]. However, whether VE and MT have the resistance to Cd-induced oxidative stress is still unknown. This study found that VE and MT could effectively protect the morphology of mitochondria and endoplasmic reticulum, restore the activities of antioxidant enzyme systems (CAT, SOD, and GSH-PX), and increase genes transcriptional expression of* CAT*,* SOD*, and* GSH-Px* in the liver cells of grass carp. Therefore, the results demonstrate that both VE and MT have the ability to resist Cd-induced oxidative stress and protect the liver.

In addition, the antioxidant efficacy of the two drugs in recovering Cd-induced damage had been detected. Both VE and MT could resist to Cd-induced oxidative stress but MT seems to have a superior effect. For example, MT appeared to be able to stabilize antioxidases activity and recover the genes expression back to normal levels more effectively than VE. This may be due to the dual role of MT as an antioxidant that eliminates ROS directly [40], as well as the capability of chelating Cd in the liver [22], which further promoted the improvement on antioxidative capacity by reducing the Cd accumulation. However, whether the combined exploit of VE and MT has a synergistic effect to enhance their antioxidant capacity, further research is needed.

## 5. Conclusions

VE and MT could protect the liver by maintaining the morphology and function of grass carp livers, recovering the antioxidases activities and elevating the antioxidase related gene transcription after Cd poisoning. Combined with our previous study [22], MT could be developed as a potential hepatinica and antidote for Cd poisoning, not only for fish but for human in the future.

## Figures and Tables

**Table 1:** Primers used for qPCR of genes in this paper.

Primers		Sequence (5′→3′)	Accession Number
*CAT*	F	GAGTTTGCGTCCTGAATCGTTG	FJ560431
	R	CCTGAGCGTTGACCAGTTTGA	
*Cu-Zn SOD*	F	GAATAAGGCTGTTTGCGTGCTA	GU901214
	R	TTCACCCGAGAGCGTCACTG	
*GSH-Px*	F	CTGCAACCAGTTCGGACATCA	EU828796
	R	TGGGCGTTCTCACCATTCA	
*β-actin*	F	CGAGCTGTCTTCCCATCCA	DQ211096
	R	TCACCAACGTAGCTGTCTTTCTG	
*18S ribosomal RNA*	F	ACCCATTGGAGGGCAAGTCT	EU047719
	R	ACCCATTGGAGGGCAAGTCT	

## Data Availability

All other data generated or analyzed in this study are included in the article. All materials are available from the corresponding author, on reasonable request.
